# Intraoperative selective arterial calcium injection test to confirm complete resection of a proinsulinoma

**DOI:** 10.1093/jscr/rjac249

**Published:** 2022-07-30

**Authors:** Akimasa Sakamoto, Kohei Ogawa, Miku Iwata, Chihiro Ito, Mikiya Shine, Takashi Matsui, Yusuke Nishi, Mio Uraoka, Takeshi Utsunomiya, Tomoyuki Nagaoka, Kei Tamura, Naotake Funamizu, Hitoshi Inoue, Katsunori Sakamoto, Mie Kurata, Yasutsugu Takada

**Affiliations:** Department of Hepato-Biliary-Pancreatic and Breast Surgery, Ehime University Graduate School of Medicine, Toon City, Ehime, Japan; Department of Hepato-Biliary-Pancreatic and Breast Surgery, Ehime University Graduate School of Medicine, Toon City, Ehime, Japan; Department of Hepato-Biliary-Pancreatic and Breast Surgery, Ehime University Graduate School of Medicine, Toon City, Ehime, Japan; Department of Hepato-Biliary-Pancreatic and Breast Surgery, Ehime University Graduate School of Medicine, Toon City, Ehime, Japan; Department of Hepato-Biliary-Pancreatic and Breast Surgery, Ehime University Graduate School of Medicine, Toon City, Ehime, Japan; Department of Hepato-Biliary-Pancreatic and Breast Surgery, Ehime University Graduate School of Medicine, Toon City, Ehime, Japan; Department of Hepato-Biliary-Pancreatic and Breast Surgery, Ehime University Graduate School of Medicine, Toon City, Ehime, Japan; Department of Hepato-Biliary-Pancreatic and Breast Surgery, Ehime University Graduate School of Medicine, Toon City, Ehime, Japan; Department of Hepato-Biliary-Pancreatic and Breast Surgery, Ehime University Graduate School of Medicine, Toon City, Ehime, Japan; Department of Hepato-Biliary-Pancreatic and Breast Surgery, Ehime University Graduate School of Medicine, Toon City, Ehime, Japan; Department of Hepato-Biliary-Pancreatic and Breast Surgery, Ehime University Graduate School of Medicine, Toon City, Ehime, Japan; Department of Hepato-Biliary-Pancreatic and Breast Surgery, Ehime University Graduate School of Medicine, Toon City, Ehime, Japan; Department of Hepato-Biliary-Pancreatic and Breast Surgery, Ehime University Graduate School of Medicine, Toon City, Ehime, Japan; Department of Hepato-Biliary-Pancreatic and Breast Surgery, Ehime University Graduate School of Medicine, Toon City, Ehime, Japan; Department of Pathology, Ehime University Graduate School of Medicine, Toon City, Ehime, Japan; Department of Hepato-Biliary-Pancreatic and Breast Surgery, Ehime University Graduate School of Medicine, Toon City, Ehime, Japan

## Abstract

Proinsulinoma is a subtype of insulinoma that is surgically curable, but localization can be difficult as these tumors are typically too small to be visualized by imaging. We report the case of a 53-year-old woman referred to our hospital with dizziness and headache. Her blood glucose level was 46 mg/dl and Whipple’s triad was present. Although her immunoreactive insulin level during hypoglycemia was in the normal range (5.0 μU/ml), the proinsulin level was elevated (408 pmol/l). Imaging examinations showed no evidence of pancreatic tumor. A preoperative selective arterial calcium injection (SACI) test showed excessive insulin secretion in the splenic artery region, which localized the proinsulinoma to the body or tail of the pancreas, and laparoscopic spleen-preserving distal pancreatectomy was performed. Intraoperative SACI test performed after tumor removal did not show excessive insulin secretion. The intraoperative SACI test appears to be useful for localization and for confirming complete resection of proinsulinoma.

## INTRODUCTION

Proinsulinoma is a rare subtype of insulinoma characterized by oversecretion of proinsulin. A recent systematic review included only 16 of these tumors [1]. Similar to insulinomas, proinsulinomas are often small in size, with a median tumor diameter of 1.2 cm reported in the systematic review [1]. It can be difficult to localize these tumors preoperatively, particularly when small [2]. Approximately 10% of insulinomas cannot be detected by any imaging modality [3]. The reported detection rates of abdominal ultrasound (US), computed tomography (CT), magnetic resonance imaging (MRI) and endoscopic ultrasound (EUS) for localization of insulinoma are 34–65%, 44–80%, 47–70% and 74–94%, respectively [2, 4, 5]. The selective arterial calcium injection (SACI) test has been established for locating insulinomas that are undetectable using other imaging techniques. The reported detection rates of the SACI test are as high as 92–100% [6].

In this report, we demonstrate the usefulness of the intraoperative SACI test to confirm the localization and complete resection of proinsulinoma.

## CASE REPORT

**Figure 1:**
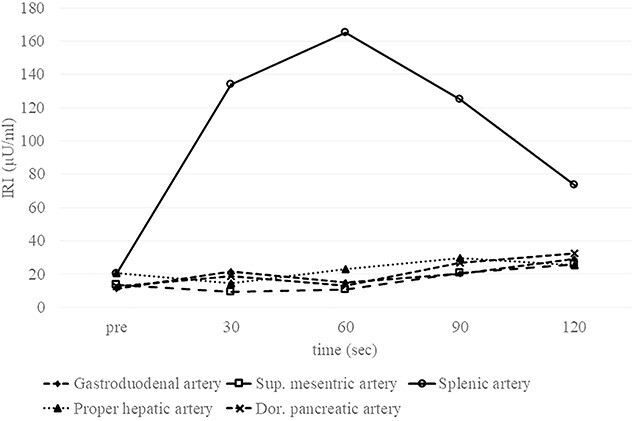
Preoperative SACI test Elevation of the IRI level by about eight-fold following injection of calcium into the splenic artery proves that the insulinoma is fed by the splenic artery. No elevation of the IRI level is observed after injection into the gastroduodenal artery, superior mesenteric artery, proper hepatic artery or dorsal pancreatic artery. Sup., superior; Dor., dorsal.

**Figure 2:**
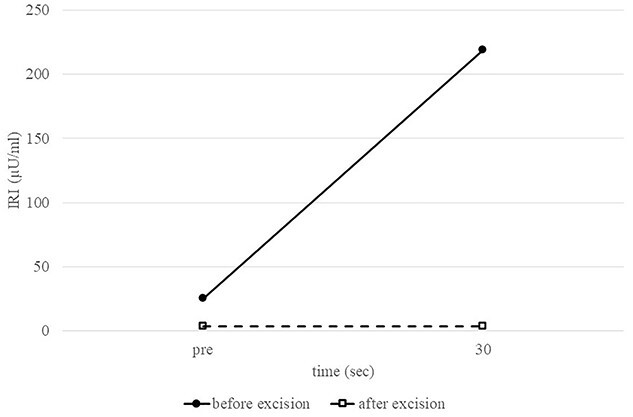
Intraoperative splenic artery SACI test. Comparison of preoperative and postoperative IRI levels after calcium injection into the splenic artery confirms the absence of excessive insulin secretion after tumor removal.

**Figure 3:**
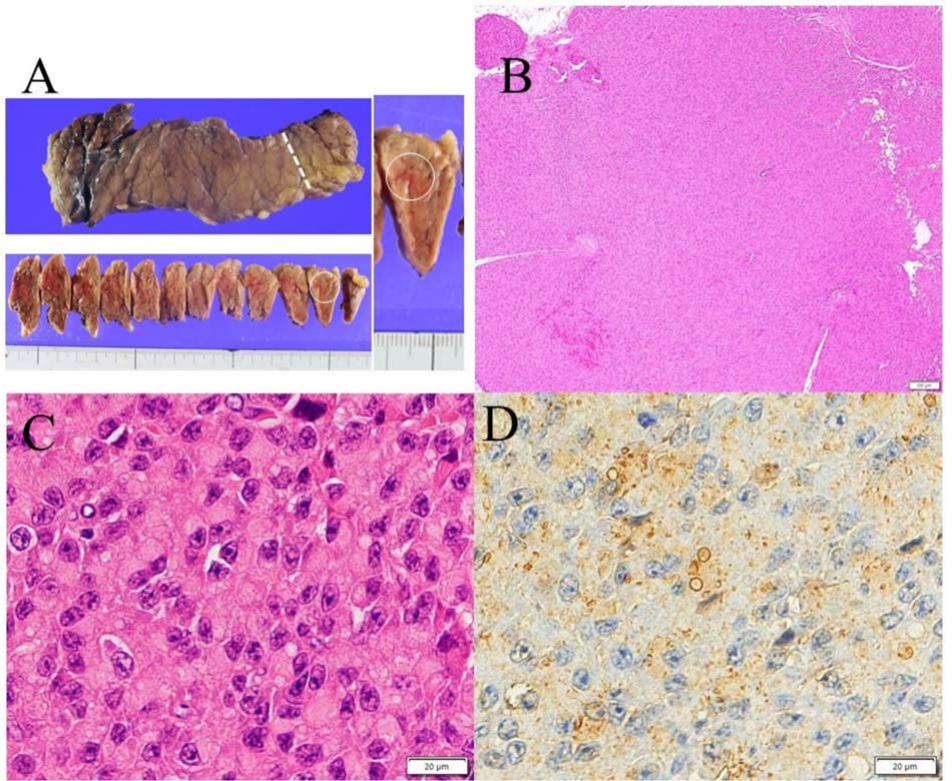
Gross appearance of excised pancreas. (**A**) Although there was no visible nodule macroscopically, tumor could be detected microscopically in the tail of the pancreas (white arrow). (**B**) Hematoxylin and eosin staining showed a solid 11-mm nodule without fibrous capsule in the tail of the pancreas. (**C**) Round to oval nuclei are seen with coarsely clumped chromatin and eosinophilic cytoplasm. (**D**) Immunostaining for insulin (Agilent, IR002) shows perinuclear Golgi pattern.

A 53-year-old woman was referred to our hospital with dizziness and headache. The physical findings were normal (height, 152 cm; weight 51 kg and body mass index, BMI, 22.1 kg/m^2^). Her blood glucose level was 46 mg/dl and Whipple’s triad was present. Fasting plasma glucose (PG) and immunoreactive insulin (IRI) levels were 44 mg/dl and 5.0 μU/ml, respectively. Although the plasma IRI level was not high enough to fulfill the classical criteria for insulinoma (Fajan’s ratio [IRI/PG] ≥ 0.3 or Turner’s ratio [IRI × 100/(PG-30) ≥ 200]), a blood test during hypoglycemia showed no suppression of C-peptide or proinsulin levels, which were 1.19 ng/ml (0.5–2.0 ng/ml) and 408 pmol/l (3–10 pmol/l), respectively. Endocrine hormones including cortisol, glucagon, thyroid hormone and growth hormone were within normal limits. Although the blood test results raised the suspicion of proinsulinoma, no tumor was detected by abdominal dynamic CT, MRI, fluorodeoxyglucose-positron emission tomography (FDG-PET), octreotide scan or EUS. In the SACI test, calcium injection into the splenic artery caused excessive insulin secretion of 164.9 μU/ml (~8 times the pre-injection level, Fig. 1), localizing the proinsulinoma in the body to the tail of the pancreas. Surgery was planned but as there was concern that the tumor might not be recognized by palpation or intraoperative US (IOUS), it was decided to perform the SACI test intraoperatively to confirm the absence of residual tumor. The SACI test was performed in a hybrid operating room for angiography. Prior to surgery, a catheter was placed in each of the celiac artery (via the femoral artery) and the hepatic vein (via the femoral vein). A pre-excision SACI test was performed to confirm an increase in the insulin level, after which the operation was started. As expected, the suspected lesion was not detected on IOUS, and laparoscopic spleen-preserving and splenic vessel-preserving distal pancreatectomy was performed. SACI test after excision of the specimen showed no excessive insulin secretion, confirming that there was no residual tumor (Fig. 2). Histopathological examination confirmed a neoplasm with a diameter of 11 mm that was immunostained for insulin in the tail of the pancreas (Fig. 3). The patient had an uneventful postoperative course and the C-peptide and proinsulin levels improved to within normal limits. To date, no recurrence of hypoglycemia has been observed.

## DISCUSSION

Proinsulin is a precursor to insulin, and consists of two chains in the insulin molecule linked together by a C-peptide [1]. Proinsulin and insulin have the same hypoglycemic activity. Although proinsulin is one-tenth as effective as insulin, oversecretion leads to hypoglycemia. In the literature, proinsulinoma is described as being rare, and most publications are case reports. Kringer *et al*. suggested that a diagnosis of proinsulinoma can be made according to the clinical presentation and an elevated proinsulin level [7]. In the present case, the patient had hypoglycemic symptoms and a proinsulin level of 408 pmol/l, which is extremely high compared with previous reports of proinsulinoma [8, 9]. Moreover, although there was no change in the IRI level after removal of the insulinoma, the proinsulin level improved, suggesting that the hypoglycemic symptoms were related to proinsulin; i.e. proinsulinoma.

Both proinsulinoma and insulinoma are treated by radical surgical resection; hence, accurate preoperative localization and intraoperative complete removal of the tumor are crucial to surgical success. Incomplete resection should absolutely be avoided because it leads to missed curative opportunity. To preserve pancreatic function, however, excessive parenchymal resection should also be avoided.

Several studies have proposed confirming complete resection of insulinoma intraoperatively such as by a quick IRI assay and intraoperative blood glucose measurement [10–12]. However, other studies reported no increase in the IRI level in some cases. An intraoperative rapid insulin assay may be ineffective in cases with no IRI elevation without selective stimulation by calcium, such as proinsulinoma. Intraoperative blood glucose measurement is a simple method for intraoperative localization [11] but may be insufficient if there are fluctuations in blood glucose levels, which can occur due to anesthesia and surgical invasion [12]. In contrast, the intraoperative SACI test can confirm complete removal of proinsulinoma by direct measurement of IRI. In the present case, the intraoperative SACI test was useful for confirming complete resection with no residual tumor. The disadvantages of the intraoperative SACI test are that it is invasive and time-consuming, but these are outweighed by its ability to confirm complete resection and thus prevent reoperation.

Proinsulinoma does not show a significant increase in the proinsulin level with calcium stimulation, in contrast to the increase in IRI level [11]. The fact that the SACI test can provide reliable localization by measuring IRI instead of proinsulin suggests the universal usefulness of this test.

## CONCLUSION

The intraoperative SACI test appears to be helpful for confirming the complete removal of proinsulinoma.

## CONFLICT OF INTEREST STATEMENT

None declared.
